# Transcription factors are potential therapeutic targets in epilepsy

**DOI:** 10.1111/jcmm.17518

**Published:** 2022-09-06

**Authors:** Qihan Sun, Wenbo Xu, Jingjing Piao, Jingyun Su, Tongtong Ge, Ranji Cui, Wei Yang, Bingjin Li

**Affiliations:** ^1^ Jilin Provincial Key Laboratory on Molecular and Chemical Genetic The Second Hospital of Jilin University Changchun China

**Keywords:** epilepsy, mechanism, transcription factors

## Abstract

Academics generally believe that imbalance between excitation and inhibition of the nervous system is the root cause of epilepsy. However, the aetiology of epilepsy is complex, and its pathogenesis remains unclear. Many studies have shown that epilepsy is closely related to genetic factors. Additionally, the involvement of a variety of tumour‐related transcription factors in the pathogenesis of epilepsy has been confirmed, which also confirms the heredity of epilepsy. In this review, we summarize the existing research on a variety of transcription factors and epilepsy and present relevant evidence related to transcription factors that may be targets in epilepsy. This information is of great significance for revealing the in‐depth molecular and cellular mechanisms of epilepsy.

## 1 INTRODUCTION

Epilepsy is a chronic brain disease characterized by transient involuntary convulsions caused by the abnormal discharge of brain neurons. Epidemiological surveys have shown that approximately 70 million patients suffer from epilepsy worldwide, with this number increasing annually by approximately 2.4 million. Affected at the societal, environmental and medical level, nearly 90% of these patients are in developing countries.^1^ There are approximately 9 million patients with epilepsy in China, giving an overall prevalence of 7.0‰, and the incidence of epilepsy has increased annually in recent years; epilepsy has become the second most common chronic disease after headache. In theory, the factors that cause abnormal signal transmission between brain nerve cells may cause epilepsy, but in most epilepsy cases, the cause is not obvious. Although there have been many studies on the pathogenesis of epilepsy, the clinical manifestations of epilepsy are diverse, and its pathogenesis is complex and has not been fully elucidated. Studies have shown that epilepsy sometimes shows a familial tendency, indicating that genetic factors have a certain influence on the formation of epilepsy.^2^ In addition, seizures in epilepsy cause continuous changes to the brain's neural network, which are reflected in changes in gene expression patterns and intra‐ and inter‐cellular signal transduction. Therefore, it is of great significance to explore the mechanism of epilepsy at the molecular level and find new molecular targets in epilepsy.

Transcription factors are a group of proteins that can bind specific DNA sequences upstream of the 5′ ends of genes to increase or block the recruitment of RNA polymerase to specific genes, thereby exerting a regulatory effect. Transcription factors contain one or more DNA‐binding domains and are characterized by polymorphism and heterogeneity; that is, one gene is regulated by multiple transcription factors, and one transcription factor regulates multiple genes. Many studies have also found that some tumour‐related transcription factors can participate in the pathogenesis of neurological diseases. However, there have been few studies on transcription factors in the study of epilepsy. In this review, we summarize the relevant studies and explore a variety of transcription factors involved in the pathogenesis of epilepsy cAMP response element‐binding protein (CREB), repressor element 1‐silencing transcription/neuron‐restrictive silencer factor (REST/NRSF), nuclear factor kappa B (NFκB), p53, p21 and Aristaless‐related homeobox (ARX). Furthermore, we propose a putative, potential epilepsy therapeutic target, Kruppel‐like factor 4 (KLF4). KLF4 is a transcription factor containing a zinc finger modified structure, similar to other zinc finger proteins, and it participates in gene expression regulation by binding to the domains of downstream target genes. Studies have shown that KLF4 can regulate the expression of CREB, NFKB, p53, p21 and other transcription factors that have been confirmed to be involved in the pathogenesis of epilepsy,^3^ and its multiple biological functions also suggest that it is closely related to epilepsy. Therefore, exploring the relationship between KLF4 and epilepsy will help to clarify the pathogenesis of epilepsy and discover new therapeutic targets.

## 2 EPILEPSY‐ASSOCIATED TRANSCRIPTION FACTORS

### 2.1 CREB

The transcription factor CREB was discovered in 1988 and can bind the cAMP response element (CRE), exerting a regulatory effect on cell transcription via the mutual conversion between phosphorylation and dephosphorylation.^4^ Numerous studies have confirmed that CREB is involved in many physiological processes, such as cell cycle regulation, neuroplasticity, learning and memory.^5^ In recent years, the role of CREB in epilepsy has gradually been revealed (Table 1). Autopsy results have shown increased expression of the *CREB* gene in the cerebral cortex of patients with temporal lobe epilepsy (TLE).^6^ CREB activation (increased phosphorylation and protein levels) was also detected in the hippocampus.^7^ Similarly, increased expression levels of CREB and phosphorylated CREB (p‐CREB) were also detected in the hippocampus of epileptic rats, and these expression levels remained high for 8 weeks.^8^ The expression of CREB in kainic acid (KA)‐induced epilepsy model mice was found to be highly consistent with that in human TLE patients, and inhibition of CREB could reduce the severity of epileptic seizures.^9^ Furthermore, a study showed that increased expression of CREB increased the excitability of hippocampal CA1 pyramidal neurons.^10^ CREB knockdown increased oxidative stress and neuronal apoptosis in TLE mice.^11^ Additionally, valproic acid treatment could reverse the overexpression of CREB in children with epilepsy.^12^ The epileptic seizure threshold and expression level of brain‐derived neurotrophic factor (BDNF) were increased in mice with low CREB levels (CREB[α∆] transgenic mice) compared with control mice.^13^ The effects of CREB and BDNF are complementary. The binding of BDNF and its receptor tropomyosin receptor kinase B (TrkB) activates CREB, improves the excitability of neurons and promotes the transmission of excitatory transmitters.^14^ CREB can also promote BDNF transcription, increase neuroplasticity and improve neurogenesis.^15^ All these findings provide a pathological basis for epileptic seizure and indicate the abnormal sprouting of mossy fibres.

**TABLE 1:** Treatment of epilepsy via the CREB signalling pathway

Therapy	Animal	Model	Mechanism and influence on epilepsy	Reference
Huazhuo Jiedu Shugan decoction	Rat	PTZ	Extends the epilepsy incubation period, improves cognition and activates the AC‐cAMP‐CREB signalling pathway	86
Salvianolic acid B	Rat	PTZ	Activates the AKT/CREB/BDNF signalling pathway, inhibits neuronal apoptosis	87
Luteolin	Rat	PTZ	Raises the seizure threshold in epilepsy, improves cognitive impairment and activates the PKA/CREB/BDNF signalling pathway	88
Thioperamide	Rat	PTZ	Inhibits epileptic seizures, improves learning and memory impairment and reverses decreased p‐CREB expression in the hippocampus	89
Enriched environment	Rat	PTZ	Reverses spatial memory impairment and the decrease in CREB expression in the hippocampus	90
Thymoquinone and vitamin C	Rat	PTZ	Exerts an anticonvulsant effect by activating the GABAB1R/CaMKII/CREB signalling pathway	91
1‐Trifluoromethoxyphenyl‐3‐(1‐propionylpiperidin‐4‐yl) urea	Rat	Lithium‐pilocarpine	Reverses neuronal damage and decreased CREB expression in the hippocampus and prefrontal cortex	92
Astaxanthin	Rat	Electrical stimulation	Improves hippocampal neuron disease and prevents decreased CREB and BDNF expression	93
Hesperidin (3,5,7‐trihydroxyflavanone 7‐rhamnoglucoside)	Zebrafish	PTZ	Suppresses epileptic seizures through the CREB/BDNF signalling pathway	94

Abbreviations: AC, adenylate cyclase; AKT, protein kinase B; BDNF, brain‐derived neurotrophic factor; CaMK, Calcium(Ca2+)/calmodulin(CaM)‐dependent ki; cAMP, cyclic adenosine monophosphate; CREB, cAMP response element‐binding protein; GABAB1R, gamma‐aminobutyric acid B1 receptor; pCREB, phosphorylated CREB; PKA, protein kinase A; PTZ, pentylenetetrazole.

Gamma‐aminobutyric acid (GABA) is an important inhibitory neurotransmitter in the central nervous system. A decreased GABA concentration and receptor dysfunction have been detected in epilepsy patients and animal models.^16^ GABA agonists have been indicated to have anti‐epileptic effects in multiple studies,^17^ and their antagonists usually cause seizures.^18^ CREB can regulate expression of the GABAA‐α1 subunit, which leads to specific changes in GABAA receptors, promoting/inhibiting epilepsy.^19^ The binding of CREB and GABAA‐α1 in the dentate gyrus was found to be enhanced in epileptic mice. Furthermore, the overexpression of CREB had a significant inhibitory effect on GABAA‐α1.^19^

**FIGURE 1:**
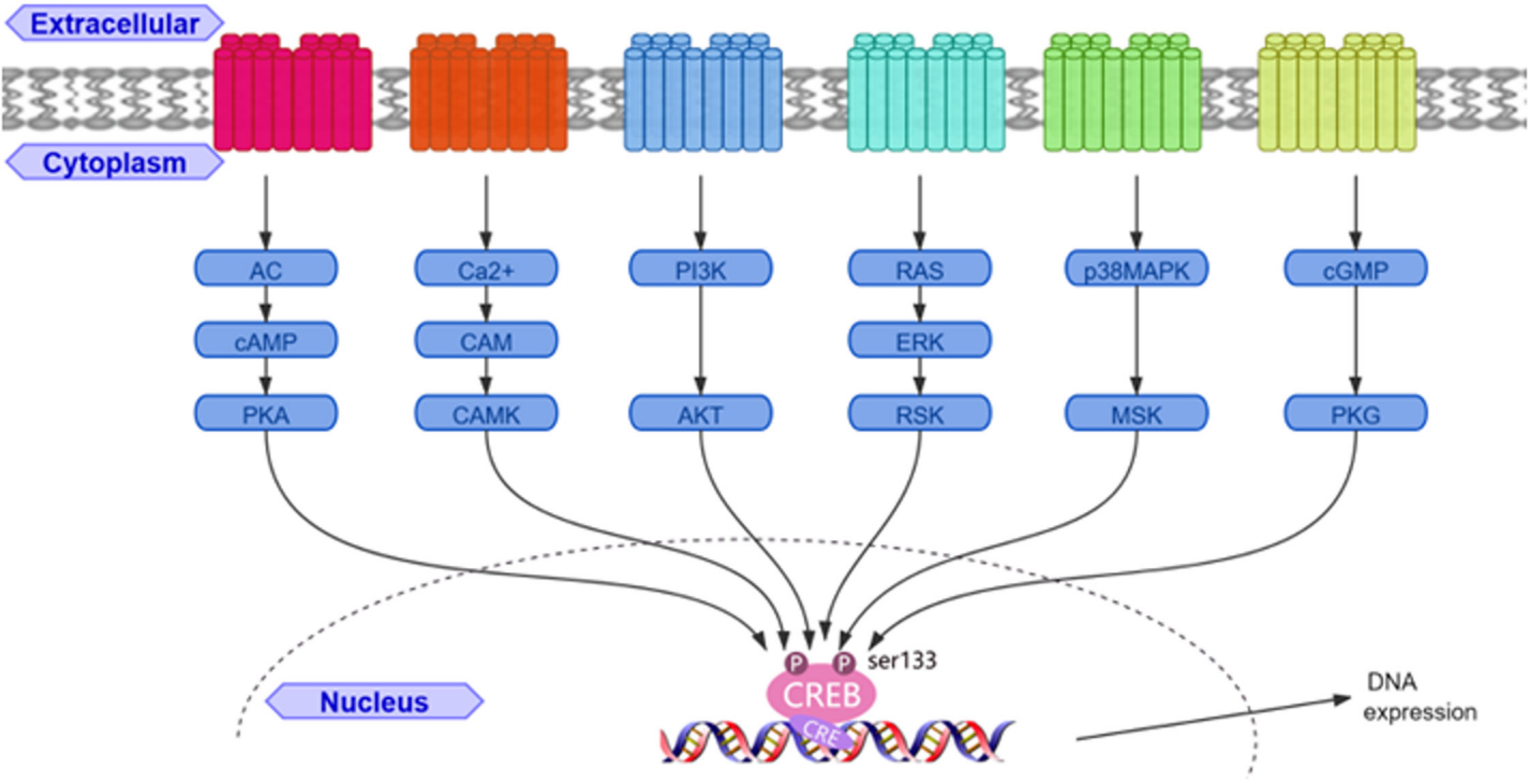
Diagram of the CREB relevant signalling pathways. AC, adenylate cyclase; AKT, protein kinase B; CAM, calmodulin; CAMK: CAM‐dependent protein kinase; cAMP, cyclic adenosine monophosphate; cGMP, cyclic guanosine monophosphate; ERK, extracellular‐regulated kinase; MSK, mitogen and stress‐activated protein kinase; p38MAPK, p38 mitogen activated protein kinases; PI3K, phosphoinositide 3‐kinase; PKA, protein kinase A; PKG, cGMP‐dependent protein kinase 1.

Numerous studies have confirmed that a variety of signalling pathways can activate CREB, phosphorylating CREB and causing it to bind the CRE region of downstream target genes to regulate DNA expression (Figure 1). At present, research on the CREB signalling pathway in epilepsy is mainly focused on AC‐cAMP‐CREB, and the potential of other CREB‐related signalling pathways in epilepsy remains to be explored.

### 2.2 REST/NRSF

REST/NRSF is a transcriptional repressor that silences target genes through epigenetic remodelling and regulates neurogenesis, differentiation and the expression of specific neuronal genes in brain development.^20^ REST was reported to regulate thousands of target genes. These genes encode neuronal receptors, ion channels, neuropeptides and synaptic proteins, and are closely related to synaptic plasticity and vesicle transport.^21^ In clinical studies, it was found that REST and BDNF gene polymorphisms can be used as markers for the diagnosis of cognitive impairment in epilepsy.^22^ However, the role of REST in epilepsy is currently controversial. Pharmacological studies have shown that both of the classic antiepileptic drugs carbamazepine and phenytoin reduce the expression of REST.^23^ REST was shown to be overexpressed in the hippocampus in patients with drug‐resistant TLE, and the degree of epilepsy was positively correlated with the expression of REST.^24^ Elevated REST levels in the hippocampus of mice preferentially inhibited approximately 10% of REST‐containing genes and promoted epilepsy.^25^ The blockade of REST prevented abnormal γ‐band oscillations and cognitive impairment after febrile convulsions and protected the integrity and function of the dentate gyrus.^26^ In a mouse model of KA‐induced epilepsy, REST blockade restored inhibited hyperpolarization‐activated cyclic adenosine monophosphate gated channel type 1 expression to a normal level.^27^ Other studies have provided evidence to the contrary. Compared with controls, in rats resistant to pentylenetetrazole **(**PTZ), the expression level of REST was increased, and the level of its downstream target gene BDNF was increased. However, the expression level of TrkB differed from that in kindled rats but was not significantly increased.^28^ This may be related to a preventive effect on epilepsy, because the knockout (KO) of TrkB, but not BDNF, was confirmed in an epilepsy kindling model to completely block the development of epilepsy.^29^ In another study, after specific REST KO in mouse forebrain excitatory neurons, the seizure onset threshold in mice with epilepsy was reduced, and mossy fibre sprouting was significantly increased.^30^ The above evidence shows that REST plays a role in inhibitory epilepsy. An increase in REST may be the body's self‐protection mechanism against epilepsy. A ketogenic diet (KD) was reported to be an effective treatment for epilepsy. A study showed that the antiepileptic effect of KD is mediated by REST,^31^ but another study came to the opposite conclusion and showed that this effect does not require REST.^32^ The dual role of REST in epilepsy may be related to microRNA‐124, which effectively blocks the upregulation of REST and regulates REST target genes to exert an antiepileptic effect. It also enhances the activation of microglia, and inflammatory cytokines play a role in promoting epilepsy.^33^ The mechanism of REST in epilepsy is complicated, and further research is needed to clarify the mechanisms of its dual effects.

### 2.3 NFκB

**FIGURE 2:**
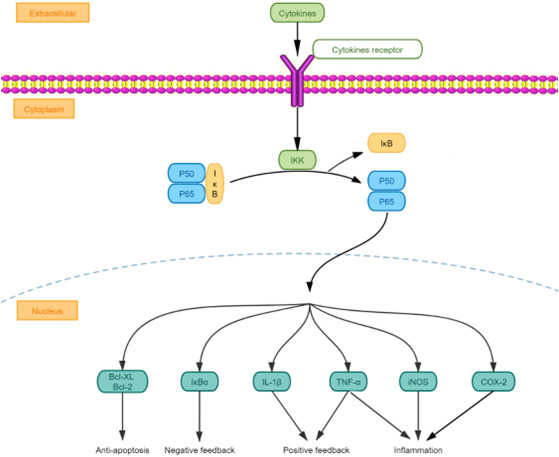
Diagram of the NFκB relevant signalling pathways. Bcl‐2, B‐cell lymphoma‐2; COX‐2, cyclooxygenase‐2; IKK, inhibitor of NFκB kinase; IL‐1β, interleukin 1 beta; iNOS, inducible nitric oxide synthase; IκB, inhibitor of NFκB; NFκB, nuclear factor kappa B; TNF‐α, tumourtumor necrosis factor‐α.

NFκB, an intracellular transcription factor required for the transcription of immunoglobulin K light chain genes in B lymphocytes, is present in almost all cells. NFκB, usually present in an inactive state, is formed by the binding of the p50‐p65 heterodimer and inhibitor of NFκB (IκB). When stimulated, it enters an activated state, induces the expression of various genes and participates in the inflammatory response. The inflammatory signalling pathway mediated by NFκB is involved in the pathogenesis of a variety of nervous system disorders.^34^ Patients with epilepsy often experience infectious or noninfectious inflammatory reactions, as well as hyperactivity of the NFκB signalling pathway caused by these reactions.^35^ A study showed abnormal expression of NFκB in the hippocampus in TLE patients.^36^ A variety of different animal models of epilepsy have also shown increased NFκB expression.^37^ Furthermore, pharmacological studies have shown that the classic antiepileptic drug phenytoin sodium could reduce the expression of NFκB in the hippocampus of model animals with penicillin‐induced epilepsy.^38^ Sodium valproate was also shown to inhibit the release of TNF‐α and IL‐6 by inhibiting NFκB activation.^39^ Target proteins induced by NFκB can also activate NFκB, thereby creating a vicious cycle in which the initial inflammatory response is amplified.^40^ This may be one of the reasons why patients with epilepsy gradually worsen and develop other comorbidities.^41^ Therefore, inhibiting the NFκB signalling pathway may become an effective method for the treatment of epilepsy. There are several ways to block the NFκB signalling pathway: inhibit NFκB phosphorylation, block NFκB nuclear localization, binding to DNA and inhibit the expression of target genes.^42^ At present, increasing evidence shows that some drugs and physical therapy methods can exert neuroprotective, antiapoptotic, anti‐inflammatory and antiepileptic effects by inhibiting the NFκB signalling pathway (Table 2 and Figure 2).

**TABLE 2:** Treatment of epilepsy via the NFκB signalling pathway

Therapy	Animal	Model	Mechanism and influence on epilepsy	Reference
Quercetin	Mouse	KA	Decreases seizure score, inhibits TNF‐α and IL‐1β release and the activation of NFκB in microglia	95
*Cnestis ferruginea* Vahl ex DC	Mouse	KA	Reduces seizure severity and downregulates COX‐2 and NFκB in the hippocampal subregions (CA1, CA2, CA1 and DG)	96
Phyllanthin	Mouse	PTZ	Improves the degree, duration and mortality of seizures; restores changes in GABA, dopamine and glutamate in kindling mice and downregulates the expression of NFκB	97
Crocin	Mouse	PTZ	Reduces the severity of seizures, improves cognitive impairment and inhibits NFκB activation	98
Dimethyl fumarate	Rat	PTZ	Reduces the degree of seizures; downregulates the expression of NFκB, Bax and caspase‐3; and increases the expression of Nrf2 and Bcl‐2	37
Progesterone	Rat	PTZ treatment after TBI	Reduces susceptibility to epilepsy; increases the protein levels of Nrf2 and HO‐1 in the hippocampus; and reduces the levels of NFκB, BDNF and Caspase 3 and the ratio of Bax/Bcl‐2	99
Electric stimulation	Rat	KA	Reduces seizures and downregulates the expression of NFκB	100
Sinomenine	Rat	KA	Reduces the degree of seizures and abnormal sprouting of mossy fibres; inhibits increased NFκB, TLR4, TNF‐α, GFAP and caspase 1	101
Berberine	Rat	KA	Reduces the frequency of seizures, reduces hippocampal inflammation (NFκB, IL‐1β and TNF‐α) and oxidative stress (reactive oxygen species, glutathione)	102
Edaravone	Rat	Lithium‐pilocarpine	Decreases the expression of IL‐1β and NFκB in the hippocampus, reduces neuronal apoptosis	103
Astaxanthin	Rat	Lithium‐pilocarpine	Improves cognitive impairment caused by epilepsy, reduces hippocampal damage and downregulates the levels of inflammatory mediators (NFκB, COX‐2, IL‐1β and TNF‐α)	104
(−)‐Epigallocatechin‐3‐gallate	Rat	Lithium‐pilocarpine	Inhibits the NFκB signalling pathway, reduces the frequency of seizures, improves cognitive impairment and reverses L‐LTP synaptic dysfunction	105
Ghrelin	Rat	Lithium‐pilocarpine	Inhibits NFκB and TNF‐α, reduces cortical nerve inflammation and neuron loss	106
MicroRNA‐494	Rat	Lithium‐pilocarpine	Inhibits the NFκB signalling pathway, hippocampal neuronal apoptosis and neuronal damage	107
*Rosmarinus officinalis* L.	Murine macrophages	Lipopolysaccharide	Prevents the activation of NFκB, reduces the expression of iNOS and COX‐2 and prevents inflammation	108
*Orthosiphon stamineus*	Zebrafish	PTZ	Acts as an anticonvulsant; reverses the upregulation of NFκB, NPY and TNF‐α	109

Abbreviations: Bax, Bcl‐2‐associated X protein; Bcl‐2, B‐cell lymphoma‐2; BDNF, brain‐derived neurotrophic factor; CA, cornu ammonis; COX‐2, cyclooxygenase‐2; DG, dentate gyrus; GABA, gamma‐aminobutyric acid; GFAP, glial fibrillary acidic protein; HO‐1, haeme oxygenase‐1; IL‐1β, interleukin 1 beta; iNOS, nducible nitric oxide synthase;KA, kainic acid; LTP, long‐term potentiation; NFκB, nuclear factor kappa B; NPY, neuropeptide; Nrf2, nuclear factor erythroid 2‐related factor 2; PTZ, pentylenetetrazole; TBI, traumatic brain injury; TLR4, toll‐like receptor 4; TNF‐α, tumour necrosis factor‐α.

### 2.4 p53 and p21

p53 and p21 are important tumour suppressor genes. p53 forms a DNA checkpoint with its downstream gene p21 and exerts a tumour‐suppressive effect. In stress and injury models, increased p53 expression levels have been shown to be related to neuronal damage, and decreased p53 expression levels have neuroprotective effects.^43^ In the PTZ‐induced kindling model, p53 showed high expression in the CA3 region of the hippocampus of rats.^44^ A similar observation was reported in the hippocampus in human patients with intractable human TLE.^45^ A variety of microRNAs, including microRNA141,^46^ microRNA128^47^ and microRNA155, can promote epilepsy through the p53 pathway. p53‐KO mice showed less cell death and damage under acute KA induction than control mice.^48^ However, in a study of long‐term p53 functional loss, p53‐deficient mice showed a more severe epileptic phenotype than controls.^49^ In addition to its requirement for apoptosis, p53 is necessary for axon growth and regeneration.^50^ p53 also plays an important role in maintaining synaptic homeostasis. p53 can regulate the stability of the glutamate receptor subunit 1 receptor in epilepsy‐induced neuronal hyperactivity through neural precursor cell expressed developmentally down‐regulated gene 4‐like (Nedd4‐2).^51^ In an epilepsy model induced by KA, the murine double minute‐2 (Mdm2)‐p53‐Nedd4‐2 signal was changed, p53 in the rat cortex was increased and susceptibility to epilepsy was increased. Furthermore, decreasing p53 could improve synchronization of the neural network.^52^

There have been few reports on p21 in epilepsy, but some studies have confirmed its relationship with epilepsy. p21 protein‐activated kinase 1 is associated with epilepsy.^53^ Valproic acid (a classic antiepileptic drug) could increase the expression of mouse p21 by changing the level of DNA methylation, thereby changing the proliferation of hippocampal cells.^54^

### 2.5 ARX

Aristaless‐related homeobox, a transcription factor involved in the development of GABAergic neurons and cholinergic neurons, is expressed in multiple brain regions related to epilepsy, including the cortex, hippocampus, striatum and basal ganglia.^55^ ARX mutations have been identified as associated with a variety of genetic diseases (X‐linked mental retardation), malformations of cortical development and epilepsy.^56^ A mouse model containing an ARX polyalanine expansion mutation showed a phenotype similar to that in humans with ARX mutation (epilepsy, learning dysfunction); furthermore, GABAergic neurons and choline were observed in the medial septum and ventral forebrain nuclei, and the specificity of functional neurons was reduced.^57^ In another study, ARX mutant mice exhibited rostral cortex migration disorders and GABAergic interneuron development defects.^58^ In addition, ARX KO mice showed disordered cortical cell proliferation^59^ and abnormal neuronal development.^60^ In terms of treatment, early developmental estradiol therapy can prevent ARX‐related infantile spasms and seizures by regulating downstream target genes of ARX.^61^

## 3 PROSPECT OF KRUPPEL‐LIKE FACTOR 4 IN EPILEPSY

**FIGURE 3:**
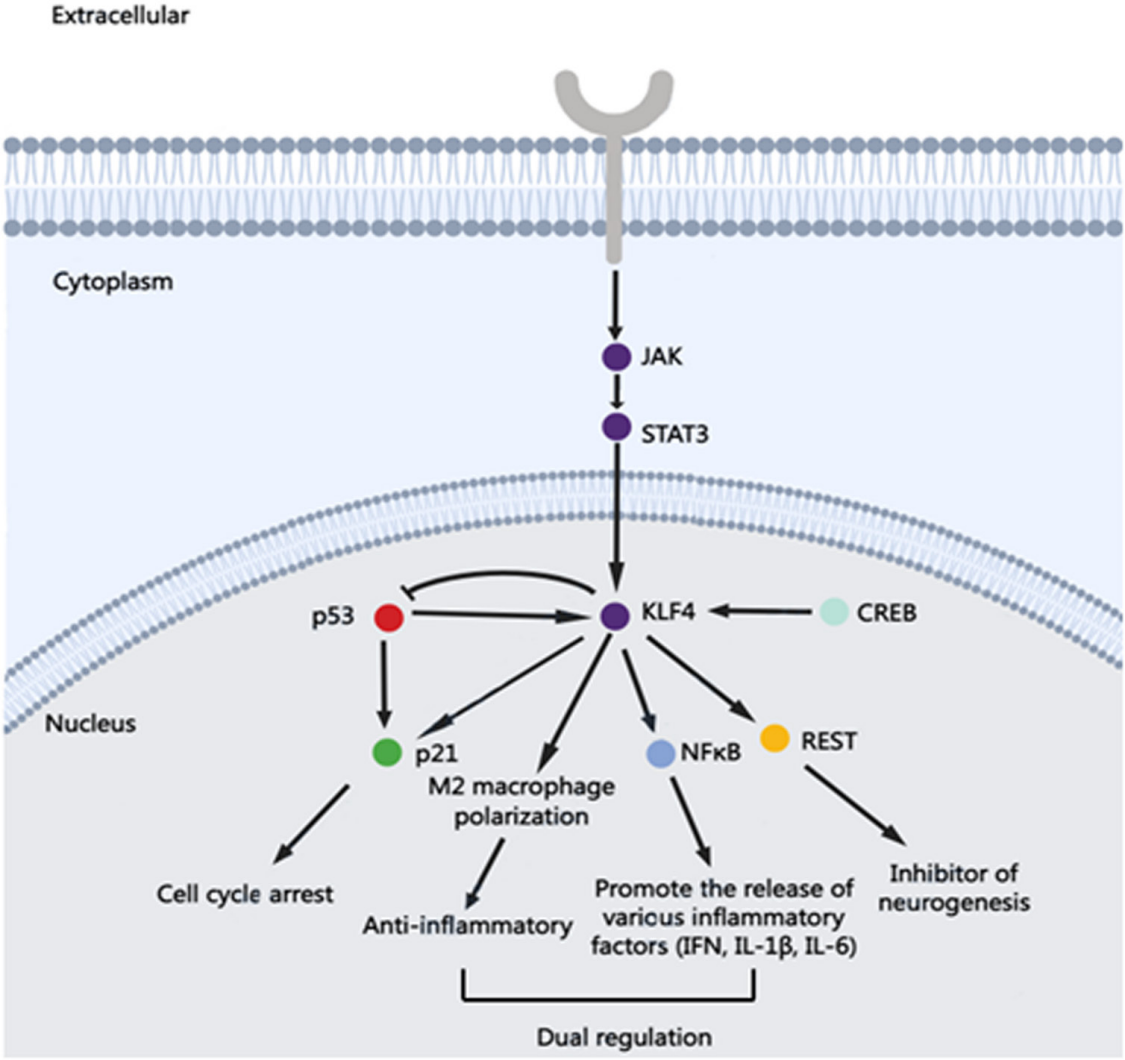
Diagram of the involvement of KLF4 in regulating the expression of multiple transcription factors. CREB, cAMP response element‐binding protein; IFN, interferon; IL‐1β, Interleukin 1 beta; IL‐6, interleukin 6; JAK, janus kinase; KLF4, Kkruüppel‐like factor 4; NFκB, nuclear factor kappa B; REST, repressor element 1‐silencing transcription; STAT3, signal transducer and activator of transcription 3.

Since the discovered epilepsy therapeutic targets cannot well explain the pathogenesis of epilepsy and cannot provide satisfactory therapeutic effects in drug‐resistant epilepsy patients, we propose a novel target based on the transcription factor level. KLF4, a member of the Kruppel‐like transcription factor protein family, is an evolutionarily conserved eukaryotic zinc finger protein transcription factor that was first found to be expressed in epithelial cells of the intestine.^62^ It plays a crucial role in many physiological processes including cell growth, proliferation, differentiation and apoptosis.^3^ KLF4 has become a hot topic in tumour research due to its dual regulatory roles (as both an oncogene and tumour suppressor gene). In recent years, successive studies have found that KLF4 is also expressed in the nervous system and involved in neural stem cell differentiation and apoptosis, oxidative stress, neuroinflammation and axon regeneration.^63^ KLF4‐mediated gene transactivation regulates multiple cellular processes through regulation of multiple post‐translational modifications in a context‐dependent manner (Figure 3). Additionally, the relationships between KLF4 and cerebral edema^64^ and Alzheimer's disease (AD),^65^ both of which are the causes of epilepsy, have been confirmed. Additionally, electroconvulsive therapy was found to increase the mRNA expression of KLF4 in patient blood.^66^ KLF4 showed different expression patterns in type I and type II focal cortical dysplasia (a cause of drug‐resistant epilepsy).^67^ The above evidence indicates that KLF4 may be involved in the pathogenesis of epilepsy, but there have been few related reports. In the next section, we focus on the involvement of KLF4 in neuroinflammation, apoptosis, neurogenesis and autophagy and provide relevant evidence to suggest exploration of the relationship between KLF4 and epilepsy. Such exploration is of great significance for finding new targets for epilepsy treatment.

### 3.1 Neuronal differentiation

Kazutoshi et al.^68^ first reported that the four transcription factors KLF4, Oct4, Sox2 and c‐Myc can jointly induce mouse fibroblasts to transform into pluripotent stem cells. A study showed that the overexpression of only KLF4 could promote the regeneration of retinal ganglion cells.^69^ Epilepsy is usually accompanied by neuronal differentiation disorders. In epilepsy, stem cells differentiation into glial cells is increased, resulting in neuron loss and damage to the neural network structure. The studies have shown that upregulation of KLF4 can induce synapse formation in hippocampal neurons.^70^ In chronic brain injury models, increasing the expression of KLF4 led to increased hippocampal neurogenesis and neuroplasticity.^71^ Furthermore, the effect of microRNA‐29a in promoting neuronal differentiation, but not glial cell differentiation, is mediated through KLF4.^72^ The regulatory effect of KLF4 on neuronal differentiation may improve the glial state of the brain in patients with epilepsy.

### 3.2 Axon growth

Abnormal sprouting of mossy fibres (axons of granular cells in the hippocampal dentate gyrus) has been confirmed by many studies as a main feature indicative of changes in brain plasticity in TLE patients. In general, the projection of mossy fibres shows directional and lamellar specificity, and abnormal sprouting caused by epilepsy forms abnormal neural circuits with peripheral neural networks.^73^ A number of studies have confirmed that KLF4 can regulate the regenerative potential of axons. Furthermore, the expression of KLF4 inhibits the growth of axons during development.^74^ In contrast, KLF4 KO was shown to promote axon growth in vivo and in vitro.^75^

### 3.3 Neuroinflammation

Alzheimer's disease and epilepsy are closely related; 10%–22% of AD patients, significantly higher than the corresponding percentage of non‐AD patients, have epilepsy.^76^ High levels of amyloid deposits in AD patients can cause epileptiform discharges.^77^ KLF4 KO was shown to improve neurotoxicity caused by β‐amyloid by downregulating the release of inflammatory factors.^78^ Microglia are another target by which KLF4 regulates inflammation. Lipopolysaccharide was found to increase the expression of KLF4 in microglia.^79^ KLF4 KO reduced the inflammatory response, which involved downregulation of a variety of inflammatory factors (TNF‐α, MCP‐1, IL‐6, iNOS and Cox‐2).^80^ Furthermore, magnolol reduced the inflammatory response in astrocytes and microglia by downregulating KLF4.^81^ The KLF4‐mediated improvement of neuroinflammation has a positive effect in epilepsy.

### 3.4 Neuronal protection

Krüppel‐like factor 4 was shown to attenuate neuronal damage in traumatic brain injury (TBI) through the P53 and JAK‐STAT3 signalling pathways^82^; the ERK5‐KLF4 signalling pathway is key to this neuroprotective effect. Activation of the ERK5/KLF4 pathway increased the expression of multiple antiapoptotic genes and the Bcl‐2/Bax ratio.^83^ In addition, miR‐212 could reduce neuronal damage in Parkinson's disease models by modulating the KLF4/Notch signalling pathway.^84^

In recent years, our research group has focused on the role of KLF4 in the occurrence, development and treatment of neurological diseases. The latest research showed that KLF4 exerts a sedative effect through crosstalk between STAT3 and p53.^85^ However, further study of the mechanism of KLF4 in epilepsy remains to be conducted.

## 4 CONCLUSION

At present, although most patients can control epileptic seizures well through surgery or drugs, the mechanism of epilepsy has not yet been elucidated. Due to the increased familial aggregation of epilepsy and increased susceptibility to epilepsy caused by genetic factors, it is still necessary to investigate its in‐depth molecular mechanisms. Transcription factors are important elements that regulate gene expression, and their relationships with epilepsy have been confirmed. Among these transcription factors, KLF4 cannot be ignored due to its involvement in the regulation of neuronal stemness. Furthermore, KLF4 may be involved in the regulation of mossy fibre sprouting and abnormal migration and integration of neurons during the formation of epilepsy. Therefore, KLF4 is expected to become a potential therapeutic target for epilepsy, providing new ideas for a more in‐depth understanding of the molecular mechanism of epilepsy.

## AUTHOR CONTRIBUTIONS

**Qihan Sun:** Writing – original draft (equal); writing – review and editing (equal). **Wenbo Xu:** Writing – original draft (equal). **Jingjing Piao:** Resources (equal). **Jingyun Su:** Resources (equal). **Ge Tongtong:** Resources (equal). **bingjin li:** Supervision (equal). **ranji cui:** Supervision (equal). **wei yang:** Conceptualization (equal); writing – original draft (equal); writing – review and editing (equal).

## FUNDING INFORMATION

This work was supported by National Natural Science Foundation of China (81871070 and 81971276). Jilin Science and Technology Agency funds in China (20200301005RQ, YDZJ202102CXJD077 and 20210204006YY). Jilin Province Medical and Health Talents (2019SCZT007，2019SCZT009 and 2019SCZT013).

## CONFLICT OF INTEREST

The authors declare no conflict of interest.

## Data Availability

This is review paper. No data, models, or code were generated or used during the study.
